# Minimally invasive and surface electroporation delivery of DNA vaccines for the induction of robust humoral immune responses against HIV antigens

**DOI:** 10.1186/1742-4690-9-S2-O9

**Published:** 2012-09-13

**Authors:** NY Sardesai, AS Khan, J McCoy, F Lin, JM Mendoza, M Yang, J Yan, N Hutnick, K Muthumani, DB Weiner, KE Broderick

**Affiliations:** 1 Inovio Pharmaceuticals, Blue Bell, PA, USA; 2 University of Pennsylvania School of Medicine, Philadelphia, PA, USA

## Background

Clinical data from the HVTN-080 study demonstrated that intramuscular electroporation (EP) delivery of PENNVAX®-B DNA vaccine and the plasmid adjuvant IL-12 generated strong antigen specific cellular immune responses in humans with nearly 90% response rate. We have now developed minimally invasive EP delivery technologies (MID-EP) to target dermal tissue and demonstrate their ability to generate strong antibody (Ab) responses in animal models with DNA antigens – including small pox, influenza, dengue – and have shown protection from viremia and lethality following challenge.

## Methods

We demonstrate MID-EP delivery of consensus HIV gp140 antigens and show the generation of cross-clade neutralizing responses in guinea pigs and rabbits. These EP enhanced humoral responses were significantly broader and higher than naked DNA delivery alone or with a protein antigen. We demonstrated NAb titers against a broad panel of 15 Tier-1 HIV viruses from Clades A-D in the range of 20 -200 measured in the Tzm-Bl neutralization assay. The magnitude but not the breadth of the responses was boosted to 20-1000 range using a MID-EP DNA prime-protein boost regimen.

## Results

We further developed a surface EP device (SEP) for the simultaneous, but spatially segregated, delivery of multi-component HIV vaccines. The SEP device operates under substantially lower voltage parameters than conventional EP devices resulting in significant improvements in tolerability. The separation of multi-component HIV vaccines avoids potential issues with plasmid interference at the transcriptional or translational levels. SEP produces Ab responses comparable to the penetrating DNAEP devices.

## Conclusion

Our results suggest that MID/SEP electroporation devices offer safe, tolerable and potent methods to administer HIV DNA vaccinations in a prophylactic clinical setting. Combined with the design of novel HIV consensus based Env antigens these DNA-EP combination vaccines are suitable for further HIV vaccine product development.

